# Occupational Hazards of Surgical Smoke and Achieving a Smoke Free Operating Room Environment: Asia-Pacific Consensus Statement on Practice Recommendations

**DOI:** 10.3389/fpubh.2022.899171

**Published:** 2022-05-26

**Authors:** Anil Ashok Heroor, Belal Bin Asaf, Suryanarayana S. V. Deo, Eric Hui-Lun Lau, Chi Wei Mok, Peter Joseph DiPasco, Pradeep Jain, Utpal Anand

**Affiliations:** ^1^Fortis Hospitals, Mumbai, India; ^2^Institute of Chest Surgery, Chest Onco Surgery and Lung Transplantation, Medanta-The Medicity, Gurugram, India; ^3^Department of Surgical Oncology, All India Institute of Medical Sciences, New Delhi, India; ^4^Department of Otorhinolaryngology, Head and Neck Surgery, The Chinese University of Hong Kong, Shatin, Hong Kong SAR, China; ^5^Division of Breast Surgery, Department of Surgery, Changi General Hospital, Singapore, Singapore; ^6^Singhealth Duke-NUS Breast Centre, Singapore, Singapore; ^7^Mercy Hospital, Mercy Clinic Surgical Specialists, St. Louis, MO, United States; ^8^Gastro-Intestinal Oncosurgery, Fortis Hospitals, New Delhi, India; ^9^Department of Surgical Gastroenterology, All India Institute of Medical Sciences, Patna, India

**Keywords:** bio-aerosols, healthcare professional (HCP), occupational hazards, operating room (OR), surgical smoke

## Abstract

**Background:**

Surgical smoke generated through energy devices may present detrimental effects on individuals present in the operating room (OR). Despite the concerns possibly associated with surgical smoke, there may be no mandatory policies that suggest protective measures and limited firm standards are committed yet to address the same.

**Aim:**

The aim of this paper is to present recommendations for surgeons and OR personnel by taking a consensus approach based on available literature and its interpretation by a multi-national panel of experts.

**Methods:**

The Asia-Pacific (APAC) group was established with the aims of reviewing literature evidence, discussing key issues regarding surgical smoke and its hazards, and offering a summary of statements in achieving a smoke-free OR environment. Eleven expert surgeons from the international APAC region were gathered with the purpose of coming to a consensus on engineering, best work-practices, and administrative controls in minimizing surgical smoke exposure. A two-phase consensus method was used to obtain opinions from the expert panel of specialists. Statements with an agreement of more than 80% were accepted.

**Findings:**

For twenty-one statements, the panel achieved consensus on 17 statements; another 5 were dropped due to lack of consensus. The consensus was obtained on statements that address the need for the implementation of administrative policies, training and awareness, standard procedure for the continued use of engineering controls, stringent work practice controls and preventive controls.

**Conclusion:**

The statements presented may guide surgeons and OR personnel in the practical management of surgical smoke safety, mitigating the risks associated with it. The consensus statement also provides a series of recommendations that can be used with other stakeholders, such as policymakers, hospital administrators and professional societies, to highlight and motivate the implementation of meaningful policies.

## Introduction

Surgical smoke is the gaseous by-product of all energy devices containing aerosols that comprise both viable and non-viable cellular material (1). Tissue vaporization *via* the use of various energy-generating devices such as electrocautery devices, laser systems, ultrasonic and advanced bipolar energy devices generates surgical smoke. Surgical smoke is composed of over 80 potentially hazardous chemicals, blood and tissue particles, bacteria and virus particles (2, 3). In addition, the mutagenic effects of the carcinogens present in the surgical smoke are also of concern (4–8). With the burgeoning use of energy devices, surgical smoke is omnipresent in the day to day life of surgeons and other medical personnel working in the operating room (OR) exposing them to a variety of hazardous substances.

Surgical plume in the OR could rightly be considered a biohazard, though this fact is often neglected. As per an occupational safety and health administration (OSHA) study, ~500,000 healthcare workers including surgeons, nurses, anesthesiologists, and surgical technicians are exposed to surgical smoke every year (9). Although regulatory agencies suggest that surgical smoke is hazardous, there are limited firm standards committed as of yet to address the inhalation hazards intrinsic to surgical smoke. Currently, there is no consensus from Asia-pacific region (APAC) regarding the potential risks of surgical smoke exposure in the OR, or regarding recommended safety precautions. So far, no substantive data on the extent to which recommendations have been implemented in practice regarding the safety of surgical smoke has been available in this region. With this context and with the emphasis of generating adequate evidence for developing clinical practice recommendations, this consensus statement summarizes the common approaches and statements provided by multi-national experts from the APAC on preventing OR personnel from the hazardous effects of surgical smoke.

### Objectives

This consensus statement aims to summarize the occupational hazards of surgical smoke and present recommendations based on current scientific evidence regarding (i) surgical smoke and health risks, (ii) infra-structure of OR, (iii) open surgery/laparoscopic surgery smoke protocols, (iv) personal protective equipment, (v) training and awareness for OR personnel, and (vi) best-practices for current available equipment. Throughout, we highlight priorities in each of these areas and present suggestions in the hope that they stimulate and guide implementation of relevant interventions on how to overcome health risks posed by surgical smoke. In addition, we identify gaps in the evidence on surgical smoke mitigation strategies and what key research questions need to be answered. These statements are based on the expert opinion of the best available evidence and provide guidance that necessitates and supports the implementation of strategies that aid the adoption of a smoke-free OR environment.

#### Why Was This Consensus Statement Developed?

Healthcare worker safety and promoting strong occupational safety measures in healthcare settings is perceivably undervalued and not regulated under direct policies. During the recent coronavirus disease 2019 (COVID-19) pandemic, significant emphasis focused on patient safety and considerations for HCP safety was often not investigated beyond necessary PPE provisions. This provides an opportunity to further explore a number of factors in creating and sustaining a culture of safety in the healthcare workplace. Studies investigating the long-term effects of exposure to surgical smoke, preventive measures, perceived hazards and associated adverse events are lacking. This consensus statement provides current recommendations about interventions that promote the establishment of a smoke-free OR environment and supports the notion of maximizing efforts to utilize reasonable measures in reducing exposure to surgical smoke. It is of utmost importance for regulatory bodies to analyze this concept so that respective policies in the context of achieving a smoke-free OR environment will be implemented.

#### Available Guidelines or Recommendations on Surgical Smoke?

Present guidelines such as the Canadian Center for Occupational Health and Safety (10), the Center for Disease Control and Prevention- National Institute for Occupational Safety and Health (CDC-NIOSH) (6), Work Health and Safety—Controlling Exposure to Surgical Plume, Ministry of Health Sydney (11), International Federation of Perioperative nurses (IFPN) (12), European Operating Room Nurses Association (EORNA) (7), CDC-NIOSH Health Hazard Evaluation Report (13), Occupational Safety and Health Act (OSHA) (14) along with other global bodies (15) were referred for their recommendations on surgical smoke, how it should be regulated, what equipment should be available, what surgical attire is able to provide safety against surgical plume, and available preventive measures to avoid consequences of hazardous effects of the surgical smoke. Although regulatory agencies suggest that surgical smoke is hazardous, no specific and elaborated standard guidelines from the APAC including National Accreditation Board for Hospitals and Healthcare Providers (NABH) from India were available in this regard. The present consensus statement provided by multi-national experts from the APAC region provides greater understanding, increases awareness of the unknown effects of surgical smoke on health, and improves the standards of practice in achieving a smoke-free OR environment.

## Methods

**Figure 1:**
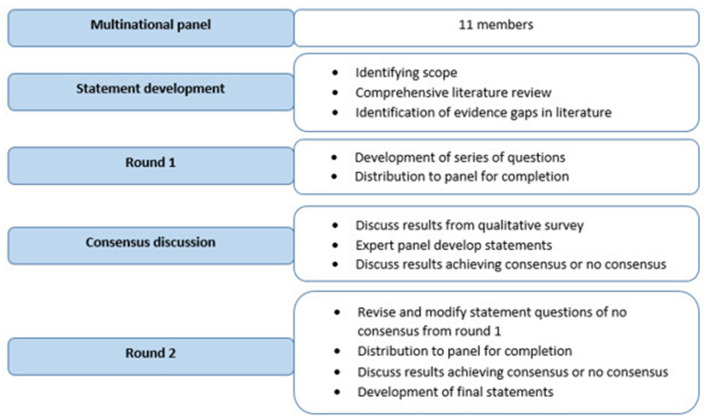
Consensus process.

For the present APAC smoke ambassador expert panel discussion, two-phase consensus rounds were used to establish consensus. This consensus statement is based on expert opinion among a panel of 11 leading surgeons from varied surgical disciplines including Gastrointestinal (GI) Oncologists and Gynecologic Oncologists. The panel included individuals from the United States, Europe, Singapore, Hong Kong, and India representing three different regions of the APAC. Figure 1 provides an overview of the consensus process used to create this APAC consensus statement.

### Statement Development

Before commencing the consensus process, a pre-read literature evidence was developed and circulated to all experts to provide an overview of available evidence and highlight important areas where the information is inadequate. A comprehensive review of the available literature was performed with terms “surgical smoke, surgical plume, bioaerosols, lung-damaging dust, surgical vapor, surgical aerosol, surgical fume, and operating room, and prevention” involving Google, Google Scholar and PubMed. Full-text publications, recommendations and guidelines published globally were searched for best practice evidence on surgical smoke-free OR environment. Information was extracted and used to develop pre-read literature evidence followed by consensus statements. Each statement was assigned the highest level of evidence available based on the review of the literature. Overall, 21 best practice statements were distributed to the panel which were grouped into 5 categories for the purposes of presentation and discussion, i.e., surgical smoke, engineering controls, work practice controls, administrative controls, and smoke-free OR.

### Round 1

The draft document containing the list of statements was distributed in July 2021 and all 11 APAC ambassador experts were invited *via* e-mail to record the responses on Microsoft office word forms. In round 1, all 11 experts replied for a response rate of 100%. The panel independently and anonymously rated the recommendations at each of the decision points from 5 categories on a five-point scale (0–4; 0-Not at all important/Not required, 1-Little important, 2-Average importance, 3-Very important, 4-Absolutely essential). Additionally, panel members were asked to mark “strongly disagree” to “strongly agree” beside each statement on a 5-item Likert scale (1 = strongly disagree, 2 = disagree, 3 = maybe, 4 = agree, 5 = strongly agree), and requested to answer single choice or multiple choice questions and provide comments where necessary. More than or equal to 80% agreement was required for the statements from the panel to accept or exclude a statement during the construction of the final guideline. The consensus conference was held virtually 5 days later, where all invited participants reviewed the evidence for surgical smoke, its hazardous effects and available preventive measures. Twenty-one statements were drafted and subjected to online consensus voting, seeking more than or equal to 80% agreement from respondents. An anonymous online poll was conducted to record participants' feedback on these questions and results were evaluated.

### Round 2

In round 2, the list of statements that did not meet the required 80% consensus from round 1 were reviewed and discussed before resending for review to all 11 members. The second round was completed by 10 experts (a 91% response rate). One expert did not respond to invitations due to reported personal needs. The same voting method was used as described for round 1. The invitation for round 2 was sent post completion of round 1 consensus. Final responses were analyzed as described for round 1, and statements not meeting expert agreement were documented as “no consensus arrived for” requiring further clinical investigation.

## Results and Consensus Outcome

In round 1, 12 out of 21 statements achieved absolute agreement (≥80% or ≤20%). Appendix 1 summarizes agreement levels of the first round of consensus for each category. In round 2, 5 out of 9 remaining statements achieved absolute agreement (≥80% or ≤20%) after panel discussion which are summarized in Appendix II. Panel members considered 17 statements they believed would most influence positive perceptions about the negative attributes of surgical smoke and were essential for a proper precautionary attitude toward surgical smoke in ORs. These statements were collated and are presented in Table 1.

**Table 1:** Key statements achieving consensus.

Surgical smoke	• Surgical smoke may have hazardous effects on health system of OR personnel • Air quality must be measured in the OR as an important safety measure • The type of surgical approach (Open, Laparoscopic, Robotic) is an important factor that may have impact on the surgical smoke exposure • The duration of exposure to surgical smoke is an absolute factor that may cause hazardous effects to OR personnel
Engineering controls	• All OR settings must implement measures like combination of general room ventilation and local exhaust ventilation (LEV) to reduce exposure to surgical smoke among perioperative team members • Installation of modular operating room (MOR) is a requirement to optimize surgical smoke safety in hospital settings
Work practice controls	• All hospital settings should implement policies on surgical attire particularly on respiratory masks • Scheduled breaks is the most practical solution that can be implemented to avoid the problem with prolonged PPE wearing • In absence of a smoke evacuation system, more stringent PPE should be used for OR personnel in HCZ (N100, PAPR, etc.) • Use of specially designed smoke evacuator designed to remove smoke near the source is the best smoke evacuation device/practice • Evaluating automatic activation with all types of energy devices is a crucial factors while selecting smoke evacuation devices
Administrative controls	• It must be a shared responsibility of hospital administrators, nursing staff, operating surgeon, and others to ensure smoke free OR environment • It must be a shared responsibility of hospital administrators, nursing staff, operating surgeon, and others to ensure compliance to surgical smoke policies and procedures post-implementation • Operating surgeon and nursing staff should be engaged in developing, reviewing and revising smoke free OR policies as necessary in hospital settings • Clinical safety officer must be a mandatory position / role in a tertiary care center • National policies should be in place on surgical smoke safety

### Surgical Smoke and Health Risks

The panel strongly agreed that surgical smoke which is found to contain numerous harmful compounds may have hazardous effects on the health of OR personnel. In a recent study, 81 OR personnel exposed to surgical smoke reported having experienced headaches, dizziness, watery eyes, coughs, and several other complications (16). This evidence demonstrates that healthcare workers should be more aware of the contents and hazards of surgical smoke and take necessary mitigation steps to lessen the risk in order to avoid long-term complications.

There was also consensus that air quality must be measured in the OR as an important safety measure. Many of the compounds found in surgical smoke are considered hazardous to human health and have been found at levels above recommended acute exposure limits set by national health organizations. The latest evidence suggests that surgical smoke generated by laser procedures is found to be five times higher (55.86 ± 2.79 μg/m^3^) than the recommended limit of 10 μg/m^3^ set by the World Health Organization (17). Based on existing literature, there appears to be no safe level of surgical smoke and evidence is lacking on the hazardous levels of exposure for surgeons and OR personnel. The panel reached a consensus about several aspects including the type of surgical approach such as Open, Laparoscopic, Robotic surgery and duration of exposure to surgical smoke as absolute factors that may cause hazardous effects to OR personnel. Surgeons and OR personnel need to carefully assess the dangers of surgical smoke, adjust techniques where reasonable, and make all reasonable efforts to protect themselves as well as the patients.

### Engineering Controls

Engineering controls, including local exhaust ventilation (LEV), represent the preferred way of a hierarchical approach to mitigate workplace hazards (18). In an experimental study, the use of LEV has been shown to reduce the airborne particles and volatile organic compounds (VOCs), mitigating the risk of surgical smoke exposure (19). Furthermore, several diverse governmental organizations and professional consensus groups recommend using engineering controls demonstrating how effective engineering control solutions can reduce the risk to healthcare workers (20–22). NABH recommends that operating rooms must be adequately ventilated and air filtered with an integrated high-efficiency particulate air (HEPA) filter (23). In spite of these recommendations, widespread use of LEV systems to remove surgical smoke is lacking. This deficit is attributable to a paucity of protocols, a lack of awareness of hazards of surgical smoke, and lack of commitment to controlling surgical smoke (24, 25). There are studies which reported that effective engineering controls in the OR are inadequate (26). Surveys of surgical smoke control practices found that engineering controls were used by fewer than half of the studied medical facilities for the reduction of surgical smoke during procedures (24, 27). It is crucial to institute a standard procedure for the continued use of engineering controls and implement best practices at facilities to reduce personal exposures to surgical smoke.

There was a uniform agreement among the panel members that a combination of general room ventilation and LEV must be implemented in all OR settings emphasizing the need for standards of procedures relating to the structure of OR. The panel also agreed that the installation of a modular operating room (MOR) is a requirement to optimize surgical smoke safety in hospital settings, though NABH states that MOR is not a mandatory requirement under any program (23). The concept of MOR construction in the occupied spaces aims to improve functionality, safety, comfort, sterility, durability and aesthetics. In addition to these sound precautions, NABH recommends a minimum of 20 total air changes per hour based on biological load and the installation of appropriate energy saving devices like heat recovery wheels, and run around pipes.

### Work-Practice Controls

The panel recommends establishing a reasonable smoke management system and adequate work practice controls. It is advised that all hospital settings should implement policies on surgical attire, particularly on respiratory masks. As per the CDC NIOSH guidelines, a properly fitted respirator (e.g., N95) must be used rather than a surgical or laser mask (6). Emerging evidence also illustrated that the high efficiency particulate air (HEPA) filters such as N95 respirator masks are capable to arrest fine particles effectively (28). The use of N95 respirator masks has been shown to provide key respiratory protection with a proven filtration efficiency rate of 99.93%, compared with disposable surgical masks (91.53%) (29, 30). In addition, the total protection factor of the N95 surgical respirator mask quantified through the simulated workplace protection factor (SWPF) was found to be SWPFtotal = 208–263 offering a higher level of protection compared to common surgical masks which provided minimal protection against surgical smoke (31).

The panel recommends appropriate protection against the particulate components of surgical smoke by implementing improved work practices, e.g., through the use of optimized scheduled breaks as the most practical solution that can be implemented to avoid the problem with prolonged PPE wearing.

As well demonstrated in experimental settings, surgical plume is found to harbor different viruses with different size ranges such as human papillomavirus, hepatitis B, infectious polio virus and HIV (32–36). Consequently, surgical smoke is feared to contain severe acute respiratory syndrome coronavirus 2 (SARS COV-2) due to its minute size and high transmissibility, however no evidence at present warrants the aerosol contamination of SARS COV-2 in surgical smoke. In this context, the possibility of SARS COV-2 transmission through cellular particles in surgical smoke cannot be ignored. Smoke evacuation systems have been distinguished as an effective way to eliminate surgical smoke, and LEV composed of wall suction with an in-line particulate filter and smoke evacuator is found to filter 99.9995% of contaminants ranging equal or >0.12 microns in diameter (37). Several factors need to be considered that affects the particulate removal capability of smoke evacuation devices including the efficiency and size of their filters, the flow rate of at least 0.012 m^3^/s, the propensity to differ both the flow rate and noise level (ideally below 60 decibels), portability, cost-effectiveness and ease of maintenance (38). Considering these factors, the panel recommends the use of smoke evacuator systems specially designed to remove smoke near the source and emphasizes the importance of evaluating automatic activation capability while selecting smoke evacuation devices. In absence of a smoke evacuation system, the panel recommends using more stringent PPE [N100, powered air-purifying respirator (PAPR)] as an alternative to minimize the exposure to surgical smoke in the high smoke content zones of the OR.

### Administrative Controls

In view of substantive evidence on the nature and extent of the risk associated with surgical smoke, a compelling need for more stringent administrative measures to limit exposure is essential. Procedures used for the elimination of surgical smoke from the OR vary widely in different settings. Several barriers have been identified that may contribute to obscuring definitive decisions about managing the risk of surgical smoke (39, 40). Hospitals are left to enact individual policies on smoke evacuation due to a lack of standards in place. In this consensus, the expert panel emphasizes implementing institutional policies, continuous supervision and training to enhance the safety of personnel and patients present in the OR.

The panel agreed that it must be a shared responsibility of hospital administrators, nursing staff, operating surgeons, and others to support the implementation of preventive strategies that aid the adoption of a smoke-free OR and to ensure compliance. Safety recommendations from the joint commission emphasize establishing and periodically reviewing policies and procedures for surgical smoke safety and control (41). According to the Occupational Health and Safety Framework Directive 89/391/EEC, all workers should be trained and informed of the hazards of surgical smoke and of preventive measures (14). In compliance with these recommendations, the panel reached a consensus that operating surgeons and nursing staff should be engaged in developing, reviewing and revising smoke-free OR policies as necessary in hospital settings. In addition, the role of clinical safety officer must be mandated in tertiary care settings. Though a number of standards organizations and agencies have weighed in on surgical smoke evacuation, there are no policies and laws that mandate the evacuation of surgical smoke from operating rooms. The panel agreed that national policies should be in place on surgical smoke safety that may enable healthcare settings to adapt to the safety practices which may influence positive impacts on perioperative staff as well as surgical patients.

### Recommendations and Summary of Evidence

The panel reached a consensus with absolutely essential or very important on a number of important areas, including education and regular training needs, equipment and people quality, administrative policies, smoke evacuation systems, and surgical smoke-free protocols. Particular attention was given relative to the optimal use of right size trocars. Regardless of the surgical approach, the fact that the use of appropriately sized trocars with good surgical practice cannot be over emphasized. A recent study compared the insertion and retention forces, and leak rates of commercially-available trocars and provided recommendations on methods to minimize exposure to pneumoperitoneum gas leakage. From the findings of this study, trocar chosen to be used should have a low insertion force, and more importantly a high retention force to minimize leakage (42). There was uniform agreement among all panelists that the appropriate selection of right size trocar could provide extra assurance in minimizing the risk of accidental pneumoperitoneum discharge and reducing gas leakage into the environment. The checklist of the key recommendations provided below is a crucial factor and integral to the development of appropriate risk management strategies in achieving a smoke-free OR environment.

However, there is limited evidence available to support the recommendations presented in Table 2. This fact highlights the importance and necessity of establishing consensus statements for theoretical concerns associated with surgical smoke, and emphasizes the need to assist OR personnel in minimizing their exposure to prevent health complications.

**Table 2:** Recommendations in achieving smoke free OR environment.

Filtered central wall room suction unit	Very important
Smoke evacuation system	Very important
N-95 respirators with or without filters	Very important
Right size trocars	Very important
Administrative policies in hospital settings	Very important
Surgical smoke free protocols	Very important
Education and awareness on hazards and effects of surgical smoke	Absolutely essential
Regular training on equipment's and maintenance	Very important
Regular training OR personnel/staff on biological hazards of filters etc. and disposing using standard precautions	Very important
Equipment quality	Absolutely essential
People quality (quality assurance and performance activities to improve compliance with surgical smoke)	Absolutely essential

*Absolutely essential = 100% consensus, Very important = ≥80% consensus*.

## Conclusion

The guidelines presented in this document will help to provide guidance for all healthcare providers to mitigate the risks and health hazards posed by exposure to surgical smoke. This consensus demonstrates the association of occupational hazards with surgical smoke, preventive controls and the need for the implementation of periodic policies. In summary, measurement of air quality in the OR, type of surgery the surgeons may opt, and duration of exposure to surgical smoke are deemed as absolute factors that may cause hazardous effects to OR personnel. A combination of general room ventilation and LEV in OR settings, and installation of a MOR in hospital settings are recommended to optimize surgical smoke safety. A multidisciplinary approach with education and regular training of staff, smoke evacuators near the source of emission, use of more stringent PPE in the absence of smoke evacuators, establishment of national policies and smoke free protocols are ideal. As we hope that further research may extrapolate data on exposure levels in ORs and other administrative measures that accrues on strategies for mitigating surgical smoke exposure, this evidence should be graded to lead to the creation of a guideline that would be acceptable to a wide audience.

## Data Availability Statement

The original contributions presented in the study are included in the article/Supplementary Material, further inquiries can be directed to the corresponding author.

## Author Contributions

All authors were responsible for the planning and scientific input of this consensus statement, contributed to the writing of this manuscript, have revised the paper critically for important intellectual content, and approved final version of manuscript.

## Funding

This work was supported by Ethicon, a part of Johnson & Johnson Private Limited.

## Conflict of Interest

The authors declare that the research was conducted in the absence of any commercial or financial relationships that could be construed as a potential conflict of interest.

## Publisher's Note

All claims expressed in this article are solely those of the authors and do not necessarily represent those of their affiliated organizations, or those of the publisher, the editors and the reviewers. Any product that may be evaluated in this article, or claim that may be made by its manufacturer, is not guaranteed or endorsed by the publisher.

## References

[1] Fan JK Chan FS Chu KM. Surgical smoke. Asian J Surg. (2009) 32:253–7. 10.1016/S1015-9584(09)60403-6 19892630

[2] Bigony L. Risks associated with exposure to surgical smoke plume: a review of the literature. AORN J. (2007) 86:1013–24. 10.1016/j.aorn.2007.07.005 18068405

[3] Benson SM Novak DA Ogg MJ. Proper use of surgical N95 respirators and surgical masks in the OR. AORN J. (2013) 97:458–67. 10.1016/j.aorn.2013.01.015 23531312 PMC7105909

[4] Chung YJ Lee SK Han SH Zhao C Kim MK Park SC. Harmful gases including carcinogens produced during transurethral resection of the prostate and vaporization: carcinogenic surgical smoke in prostate. Int J Urol. (2010) 17:944–9. 10.1111/j.1442-2042.2010.02636.x 20880073

[5] Gatti JE Bryant CJ Noone RB Murphy JB. The mutagenicity of electrocautery smoke. Plast Reconstr Surg. (1992) 89:785–6. 10.1097/00006534-199205000-00002 1561248

[6] Centres for Disease Control Prevention (CDC). The National Institute for Occupational Safety and Health (NIOSH). Surgical smoke. Available online at: https://www.cdc.gov/niosh/topics/healthcarehsps/s moke.html (accessed September 9, 2021).

[7] European Operating Room Nurses Association (EORNA). Recommendation on Prevention Protection of Surgical Plume. (2018). Available online at: https://eorna.eu/wp-content/uploads/2019/09/ Prevention-and-Protection-of-Surgical-Plume-PNCEORNA.pdf (accessed September 8, 2021).

[8] Association of Peri Operative Registered Nurses (AORN). Management of Surgical Smoke-Toolkit. Available online at: https://www.aorn.org/guidelines/clinicalresources/tool-kits/non-member-tool-kits/managementof-surgical-smoke-tool-kit-nonmembers (accessed September 8, 2021).

[9] OSHA. Laser/Electrosurgery Plume. Available online at: https://www.osha.gov/SLTC/laserelectrosur geryplume/index.html (accessed September 6, 2021).

[10] Canadian Centre for Occupational Health Safety. Laser Plumes—Health Care Facilities. (2014). Available online at: http://www.ccohs.ca/oshanswers/phys_agents/laser_plume.html (accessed September 10, 2021).

[11] Ministry Ministry of Health New South, Wales, Australia. Work Health and Safety - Controlling Exposure to Surgical Plume. (2015). Available online at https://www1.health.nsw.gov.au/pds/ActivePDSDocuments/GL2015_002.pdf (accessed September 9, 2021).

[12] IFPN (International Federation of Perioperative Nurses). Guideline on Risks, Hazards, and Management of Surgical Plume. (2015). Available online at: http://www.jona.gr.jp/Surgical_Plume_-_Risks_Hazards_and_Management.pdf (accessed September 11, 2021).

[13] CDC-NIOSH. Health Hazard Evaluation Report No. 2001-0066-3019. Available online at: https://www.cdc.gov/niosh/docs/hazardcontrol/hc11.html (accessed September 8, 2021).

[14] OSHA. Directive 89/391/EEC of 12 June 1989 on the Introduction of Measures to Encourage Improvements in the Safety and Health of Workers at Work—OSH ‘Framework Directive’. Available online at: https://osha.europa.eu/de/legislation/directives/the-osh-framework-directive/1 (accessed September 10, 2021).

[15] CSA, Standard Z305,.13-13 (R2020). Plume Scavenging in Surgical, Diagnostic, Therapeutic, and Aesthetic Settings. Available online at: https://webstore.ansi.org/preview-pages/CSA/preview_2422790.pdf (accessed September 10, 2021).

[16] Ilce A Yuzden GE Yavuz van Giersbergen M. The examination of problems experienced by nurses and doctors associated with exposure to surgical smoke and the necessary precautions. J Clin Nurs. (2017) 26:1555–61 10.1111/jocn.13455 27345749

[17] Jones LCR Parry M Britton J Tyrer JR. Engineering controls for surgical smoke in laser medical handpieces. J Laser Appl. (2021) 33:022007. 10.2351/7.0000360

[18] Manuele FA. Risk assessment and hierarchy of control. Prof Safety. (2005) 50:33–9.

[19] Lee T Soo JC LeBouf RF Burns D Schwegler Berry D Kashon M. Surgical smoke control with local exhaust ventilation: experimental study. J Occup Environ Hyg. (2018) 15:341–50. 10.1080/15459624.2017.1422082 29283318 PMC6460469

[20] Association of PeriOperative Registered Nurses (AORN). Standards, Recommended Practices, and Guidelines. Denver, CO: AORN Inc. (2014). Recommended Practices for Laser Safety in Perioperative Practice Settings. p. 141–54.

[21] Association of Surgical Technologists (AST). AST Standards of Practice Use of Electrosurgery. (2012). Available online at: https://www.ast.org/uploadedFiles/Main_Site/Content/About_Us/Standard%20Electrosurgery.pdf (accessed September 10, 2021).

[22] Emergency Care Research Institute (ECRI) (2007),. Laser Use and Safety. Healthcare Risk Control. Vol. 1. Plymouth Meeting, PA. Available online at: https://www.ecri.org/search-results/member-preview/hrc/pages/surgan17 (accessed September 10, 2021).

[23] Nabh.co. Revised Guidelines for Air Conditioning in Operation Theatres. (2018). Available online at: https://www.nabh.co/images/pdf/RevisedGuidelines_AirConditioning_OT2018-final.pdf (accessed September 8, 2021).

[24] Steege AL Boiano JM Sweeney MH. Secondhand smoke in the operating room? Precautionary practices lacking for surgical smoke. Am J Ind Med. (2016) 59:1020–31. 10.1002/ajim.22614 27282626 PMC5069165

[25] Ball K. Compliance with surgical smoke evacuation guidelines: implications for practice. AORN J. (2010) 92:142–9. 10.1016/j.aorn.2010.06.002 20678603

[26] Van Giersbergen MY Alcan AO Kaymakci S Ozsaker E Dirimese E. Investigation of surgical smoke symptoms and preventive measures in Turkish operating rooms. Int J Health Sci Res. (2019) 9:138–44.

[27] Edwards BE Reiman RE. Results of a survey on current surgical smoke control practices. AORN. (2008) 87:739–49. 10.1016/j.aorn.2007.11.001 18395019

[28] Georgesen C Lipner SR. Surgical smoke: risk assessment and mitigation strategies. J Am Acad Dermatol. (2018) 79:746–55. 10.1016/j.jaad.2018.06.003 29902546

[29] Edwards BE Reiman RE. Comparison of current and past surgical smoke control practices. AORN J. (2012) 95:337–50. 10.1016/j.aorn.2011.07.019 22381553

[30] Lu W Zhu XC Zhang XY Chen YT Chen WH. Respiratory protection provided by N95 filtering facepiece respirators and disposable medicine masks against airborne bacteria in different working environments. Zhonghua Lao Dong Wei Sheng Zhi Ye Bing Za Zhi. (2016) 34:643–46. 27866538 10.3760/cma.j.issn.1001-9391.2016.09.002

[31] Gao S Koehler RH Yermakov M Grinshpun SA. Performance of facepiece respirators and surgical masks against surgical smoke: simulated workplace protection factor study. Ann Occup Hyg. (2016) 60:608–18. 10.1093/annhyg/mew006 26929204 PMC7109898

[32] Alp E Bijl D Bleichrodt RP Hansson B Voss A. Surgical smoke and infection control. J Hosp Infect. (2006) 62:1–5. 10.1016/j.jhin.2005.01.014 16002179

[33] Johnson GK Robinson WS. Human immunodeficiency virus-1 (HIV-1) in the vapors of surgical power instruments. J Med Virol. (1991) 33:47–50. 10.1002/jmv.1890330110 1901908

[34] Liu Y Song Y Hu X Yan L Zhu X. Awareness of surgical smoke hazards and enhancement of surgical smoke prevention among the gynecologists. J Cancer. (2019) 10:2788–99. 10.7150/jca.31464 31258787 PMC6584931

[35] Kwak HD Kim S-H Seo YS Song KJ. Detecting hepatitis B virus in surgical smoke emitted during laparoscopic surgery. Occup Environ Med. (2016) 73:857–63. 10.1136/oemed-2016-103724 27484956

[36] Sawchuk WS Weber PJ Lowy DR Dzubow LM. Infectious papillomavirus in the vapor of warts treated with carbon dioxide laser or electrocoagulation: detection and protection. J Am Acad Dermatol. (1989) 21:41–9. 10.1016/S0190-9622(89)70146-8 2545749

[37] Schultz L. An analysis of surgical smoke plume components, capture, and evacuation. AORN J. (2014) 99:289–98. 10.1016/j.aorn.2013.07.020 24472591

[38] Fencl JL. Guideline implementation: surgical smoke safety. AORN J. (2017) 105:488–97. 10.1016/j.aorn.2017.03.006 28454614

[39] Barrett WL Garber SM. Surgical smoke: a review of the literature. Is this just a lot of hot air? Surg Endosc. (2003) 17:979–87. 10.1007/s00464-002-8584-5 12640543

[40] Champault G Taffinder N Ziol M Riskalla H Catheline JM. Cells are present in the smoke created during laparoscopic surgery. Br J Surg. (1997) 84:993–5. 10.1002/bjs.1800840724 9240145

[41] The Joint Commission. Quick Safety, Issue 56: Alleviating the Dangers of Surgical Smoke. Available online at: www.jointcommission.org/resources/news-and-multimedia/newsletters/newsletters/quick-safety/quick-safety-issue-56/quick-safety-issue-56/ (accessed September 10, 2021).

[42] Cepress JM Cummings JF Ricketts CD Clymer JW Tommaselli GA. Comparison of trocar performance in consideration of the COVID-19 pandemic. Med Devices Diagn Eng. (2020) 5:1–7. 10.15761/MDDE.1000129

