# Supplementary material for: Occupational Hazards of Surgical Smoke and Achieving a Smoke Free Operating Room Environment: Asia-Pacific Consensus Statement on Practice Recommendations
Source: Front Public Health. 2022 May 26;10:899171. doi: 10.3389/fpubh.2022.899171 (PMC9178078; doi:10.3389/fpubh.2022.899171)
Supplement: Supplementary file 1 [file Table_1.DOCX]

**Supplementary material:**

**Appendix I:**

| **Category** | **Questions** | **% Consensus** | **Decision at first round** |
| --- | --- | --- | --- |
| Surgical  Smoke | 1 Surgical smoke may have hazardous effects on health system of OR personnel? | 91% | Agreement |
|  | 2 As an important safety measure, air quality should be measured in the OR? | 91% | Agreement |
|  | 3. According to you, factors that have impact on the surgical smoke exposure? (Multiple choice question)  A. The type of surgical approach (Open, Laparoscopic, Robotic) B. The duration of exposure | 91%,  100% | Agreement |
| Engineering Controls | 4. All OR settings must implement measures like combination of general room ventilation and local exhaust ventilation (LEV) to reduce exposure to surgical smoke among perioperative team members | 100% | Agreement |
|  | 5.Do you agree that the installation of MOR must be a mandatory requirement to optimize surgical smoke safety in hospital settings | 73% | No Agreement |
| Work-practice Controls | 6. Do you agree that in absence of a smoke evacuation system, more stringent PPE should be used for OR personnel in HCZ (N100, PAPR, etc.) | 64% | No Agreement |
|  | 7. All hospital settings should implement policies on surgical attire particularly on respiratory masks? | 91% | Agreement |
|  | 8. Evidence suggests prolonged PPE wearing results in certain complications including hypoventilation resulting in headache, dizziness, increase in breathing frequency, cardiovascular effects (e.g., diminished cardiac contractility, vasodilation of peripheral blood vessels), reduced tolerance, drop in oxygen saturation levels, skin allergy/dermatitis, acne and impaired cognition etc. A. Scheduled breaks | 82% | Agreement |
|  | 9. What do you consider is the best smoke evacuation device/Practice? A. Use of Specially designed smoke evacuator designed to remove smoke near the source | 91% | Agreement |
|  | 10. Factors to be evaluated while selecting smoke evacuation devices (Multiple choice) A. Automatic activation with all types of energy devices | 91% | Agreement |
|  | 11. Please rate your level of concern in using a smoke evacuation device regarding the following aspects (0 to 4 , 0 – not at all, 4- highest) |  |  |
|  | Excessive noise | 45% | No Agreement |
|  | Equipment maintenance issue | 45% | No Agreement |
|  | Large bulky devices | 28% | No Agreement |
|  | Resistance from clinical staff | 36% | No Agreement |
|  | 12. Special individual protective measures such as eye protection and high-filtration masks are unnecessary when adequate smoke evacuation systems are maintained? | 9% | Agreement |
|  | 13. Plastic / disposable trocars should be used to minimize escape of surgical smoke in laparoscopic surgery? | 45% | No Agreement |
|  | 14. There are conflicting views on the use of smoke evacuators; some guidelines suggest that smoke evacuators should be used in every OR where surgical smoke is generated while others recommend its use where there is considerable smoke or else recommend using room wall suction with considerable room air ventilation, what do you recommend?  A) Use of smoke evacuators in every OR where surgical plume is generated; | 73% | No Agreement |
| Administrative controls | 15. How often should sessions be conducted in a hospital to ensure awareness about surgical smoke and potential hazards?  A) Biannually | 45% | No Agreement |
|  | 16. Who should be responsible to ensure smoke free operating room?  A) Shared responsibility | 73% | No Agreement |
|  | 17. Who should be responsible for compliance to surgical smoke policies and procedures post-implementation? 1. Shared responsibility of a combination of all | 82% | Agreement |
|  | 18. Who should be engaged in developing, reviewing and revising smoke free OR policies as necessary in hospital settings (multiple choice)? 1. Operating surgeon, 2. Nursing staff | 100%, 82% | Agreement |
|  | 19. Clinical safety officer to be a mandatory position / role in a tertiary care Centre? | 91% | Agreement |
|  | 20. National policies should be in place on surgical smoke safety | 55% | No Agreement |
| Smoke Free OR | 21.Your recommendations on achieving smoke free OR |  |  |
|  | Filtered central wall room suction unit | 82% | Agreement |
|  | Smoke evacuation system | 82% | Agreement |
|  | N-95 respirators with or without filters | 82% | Agreement |
|  | Right size trocars | 82% | Agreement |
|  | Add-on filters to cannula valve | 73% | No Agreement |
|  | Administrative policies in hospital settings | 82% | Agreement |
|  | Appointment of statutory members in hospital settings for surgical smoke safety | 64% | No Agreement |
|  | Surgical smoke free protocols | 64% | No Agreement |
|  | Education and awareness on hazards and effects of surgical smoke | 100% | Agreement |
|  | Regular training on equipment’s and maintenance | 91% | Agreement |
|  | Regular training OR personnel/staff on biological hazards of filters etc. and disposing using standard precautions | 91% | Agreement |
|  | Equipment Quality | 100% | Agreement |
|  | People Quality (quality assurance and performance activities to improve compliance with surgical smoke) | 100% | Agreement |

**Appendix II:**

| **Category** | **Questions** | **% Consensus** | **Decision at second round** |
| --- | --- | --- | --- |
| Engineering Controls | 5. Do you agree that the installation of MOR is requirement to optimize surgical smoke safety in hospital settings | 80% | Agreement |
| Work-practice controls | 6. Do you agree that in absence of a smoke evacuation system, more stringent PPE should be used for OR personnel in HCZ (N100, PAPR, etc.) | 80% | Agreement |
|  | 11. Please rate your level of concern in using a smoke evacuation device regarding the following aspects (0 to 4 , 0 – not at all, 4- highest) |  |  |
|  | Excessive noise | 70% | No Agreement |
|  | Equipment maintenance issue | 40% | No Agreement |
|  | Large bulky devices | 50% | No Agreement |
|  | Resistance from clinical staff | 40% | No Agreement |
|  | 13. Can disposable trocars minimize escape of surgical smoke in laparoscopic surgery as compared to reusable trocars? | 40% | No Agreement |
|  | 14. There are conflicting views on the use of smoke evacuators; some guidelines suggest that smoke evacuators should be used in every OR where surgical smoke is generated while others recommend its use where there is considerable smoke or else recommend using room wall suction with considerable room air ventilation, what do you recommend?  A) Use of smoke evacuators in every OR where surgical plume is generated; | 70% | No Agreement |
| Administrative controls | 15. How often should sessions be conducted in a hospital to ensure awareness about surgical smoke and potential hazards?  A) Biannually | 70% | No Agreement |
|  | 16. Who should be responsible to ensure smoke free operating room?  A) Shared responsibility | 80% | Agreement |
|  | 20.National policies should be in place on surgical smoke safety | 90% | Agreement |
| Smoke Free OR | 21.Your recommendations on achieving smoke free OR |  |  |
|  | Add-on filters to cannula valve | 60% | No Agreement |
|  | Appointment of statutory members in hospital settings for surgical smoke safety | 60% | No Agreement |
|  | Surgical smoke free protocols | 80% | Agreement |
